# Determinants of COVID-19 vaccine acceptance and access among people experiencing homelessness in Germany: A qualitative interview study

**DOI:** 10.3389/fpubh.2023.1148029

**Published:** 2023-03-24

**Authors:** Julianna Grune, Darius Savelsberg, Marta Kobus, Andreas K. Lindner, Wolfram J. Herrmann, Angela Schuster

**Affiliations:** ^1^Institute of General Practice and Family Medicine, Charité - Universitätsmedizin Berlin, Corporate Member of Freie Universität Berlin, Humboldt-Universität zu Berlin and Berlin Institute of Health, Berlin, Germany; ^2^Institute of International Health, Charité - Universitätsmedizin Berlin, Corporate Member of Freie Universität Berlin, Humboldt-Universität zu Berlin and Berlin Institute of Health, Berlin, Germany

**Keywords:** homelessness, prevention, vaccine acceptance, vaccine hesitancy, vaccine access, COVID-19, primary care, access to health care

## Abstract

**Introduction:**

People experiencing homelessness face lower life expectancy, higher prevalence of somatic and mental diseases and a more difficult access to healthcare compared to people in secure living. During the COVID-19 pandemic transmission rates were higher among people experiencing homelessness and preventive public health measures were not properly adapted to the specific needs of people experiencing homelessness. Thus, goal of our study was understanding the determinants of acceptability and access of the COVID-19 vaccine.

**Materials and methods:**

We conducted a qualitative interview study with twenty guideline interviews with adult people currently experiencing homelessness in Berlin, Germany (August 2021 – April 2022). Participants were approached in a purposive sampling strategy. The interviews were analyzed with qualitative content analysis according to Mayring.

**Results:**

Acceptance and attitude toward the COVID-19 vaccine is influenced by confidence in the vaccine as well as in the political and healthcare system, the individual COVID-19 risk perception and sense of collective responsibility. Overall, the acceptance of the vaccine was high among our participants. Facilities offering low threshold COVID-19 vaccines for people experiencing homelessness were perceived as helpful. Language barriers and the need for identity documents were major barriers to access the COVID 19 vaccine.

**Discussion:**

People experiencing homelessness are a marginalized and vulnerable group often underrepresented in the public and scientific discourse. During the COVID-19 pandemic, preventive public health measures, including the COVID-19 vaccine, failed to consider specific needs of people experiencing homelessness. Multidimensional strategy to enhance inclusive healthcare are needed to improve access and to reduce discrimination and stigmatization.

## 1. Introduction

Homelessness is a multidimensional social and health state, often caused by a complex network of individual and structural circumstances (1).

The *European Typology of Homelessness and housing exclusion* (ETHOS) developed by the non-governmental organization *European Federation of National Organizations Working with the Homeless* (FEANTSA) uses the following categories to cover possible living situations subsumed as homelessness: *rooflessness* (living without shelter of any kind), *houselessness* (having a temporary place to sleep), *living in insecure housing* (living with the threat of eviction or domestic violence) and *living in inadequate housing* (for instance in caravans, on illegal campsites) (2). In this article we focus on people experiencing rooflessness as well as people experiencing houselessness and refer to them as people experiencing homelessness (PEH).

According to the *National Federation for the Homeless* (Bundesarbeitsgemeinschaft Wohnungslosenhilfe, BAG W), approximately 417.000 houseless people lived in Germany in 2020. Included in this number were around 41.000 roofless people (3).

Existing studies show stark health discrepancies of PEH when compared to people in secure living situations. For instance, a systematic review from 2020 reveals a higher prevalence of cardiovascular, musculoskeletal, and respiratory diseases among PEH compared to the population in secure housing in Germany (4). A meta-analysis from 2017 draws a similar picture: mental health problems among PEH are considerably higher as compared to people in secure housing. The major share of mental health burden can be attributed to alcohol dependency and substance use disorders, further anxiety disorders, affective disorders, drug dependence, and major depression (5). Similar results were described in the German-based National Survey on Psychiatric and Somatic Health of Homeless Individuals (6). Homelessness is not only associated with a higher prevalence of somatic and mental illness, but also with higher mortality rates. According to a literature review comparing data on mortality from the United States, Canada, Europe, Asia, and Australia, PEH are three to four times more likely to die prematurely than the general population and their life expectancy is reduced by 30 years (7).

At the same time, PEH face major barriers when seeking health care (6, 8). A qualitative study from Spain identified administrative, personal, and medical-professional barriers for PEH, demonstrating inequities in accessing healthcare. Personal barriers were based on experiences of poor service, discrimination, or unaffordable treatment (9). In a Canadian study, more than one-third of the included PEH reported unmet health needs (10). According to a survey from Hamburg (Germany), uninsured PEH (one-third of the included individuals), reported fewer physician visits, again indicating a lack of affordability of health care (11). Matching those findings, a facility enabling access to health care for people without health insurance in Berlin reports 50% of their clients to be home- or roofless (12).

In times of the COVID-19 pandemic general information, health regulations or disease control measures were often delivered online. This “digital gap” leads to reduced access of PEH to preventive services (e.g., vaccination) and treatment which is enhanced by socio-economical barriers (13). Another barrier lies in the general practitioners (GPs) themselves. As shown in qualitative research among GPs in the United Kingdom, barriers to providing health service for PEH included insufficient training and inadequate consultation competences to address special needs of PEH (14). Further, PEH repeatedly experience stigmatization when accessing healthcare. Those experiences of stigma and shame may lead to avoidance of healthcare facilities (15, 16).

Access to health care is a human right (17) and is a frequently discussed and universal concern when it comes to equity in health. A broader approach to understanding access was given by Penchansky et al.: they describe access “*as a concept representing the degree of “fit” between the clients and the system”* including five different dimensions: *availability* (balanced supply and demand), *accessibility* (health service is within reach to the client in reasonable travel time and distance), *adequacy* or *accommodation* [fitting opening hours, appointment systems and facility structures (e.g., wheelchair access)], *affordability* (in means of financial and incidental costs for service providers and clients) and *acceptability* (relation between perception of social and cultural concerns of the provider and client) (18). Saurman modified that concept of access by adding a sixth dimension: *awareness*. As all other dimensions, *awareness* can be implied in both ways – meaning that it is important for health services to provide adequate information for possible clients in an appropriate way but also underline the significance of health services being aware of local context and population needs (19).

Beyond difficult access to preventive infection control measures PEH’s worse health state increased the likelihood to experience a severe course of COVID-19. Especially cardiovascular and respiratory diseases, disproportionally frequent among PEH increase the risk of severe COVID-19. PEH are confronted with higher transmission risks due to sleeping rough, lack of isolation possibilities in emergency or temporary shelters, and barriers to accessing adequate (health-)care and information (13, 20, 21). In addition to that, some non-pharmaceutical interventions – such as staying at home (without having a home), social distancing, and increased hygiene – are simply almost impossible to realize for PEH.

Vaccines are an important cost-effective public health measure (22) and can reduce incidences of different diseases (23). Vaccines work at both the individual and community levels since high vaccine coverage induces protection for the whole community and not only for vaccinated individuals (23). Therefore, vaccination programs and their success depend on a high uptake level. Increasing incidences of vaccine-preventable diseases like measles (24) and the not yet achieved eradication of poliomyelitis (25) have been linked to under or non-vaccinated communities (23). One factor reducing high vaccine uptake levels is vaccine hesitancy. Vaccines are very important in the context of homelessness: PEH are a particularly vulnerable group because their impaired health status compared to the general population and their difficulties in accessing the health care system. In this context, lower vaccination rates among PEH as compared to the general population is worrisome (26, 27). It is therefore of great importance to enhance the inclusion of PEH in vaccination strategies (28).

To understand the very complex composition of factors leading to vaccine-hesitant behavior, different approaches to examining determinants of vaccine hesitancy have been developed. Betsch et al. developed the *5C psychological antecedents of vaccination*, giving insights into individual and psychological factors. The 5C consists of five different dimensions influencing vaccine behavior: *confidence* (trust in the effectiveness of the vaccine, the delivering system, and the motivation of policy-makers), *complacency* (degree of risk perception related to a specific diseases), *constraints* (circumstances as physical availability, geographical accessibility, ability to understand consequences of a disease), *calculation* (engagement in information searching and evaluating pro and cons) and *collective responsibility* (will to protect others by vaccinating oneself) (29).

Monitoring COVID-19 vaccine uptake in Germany, the studies COSMO and COVIMO both identified safety concerns, low-risk perception of COVID-19, and distrust as the main reasons inhibiting the willingness to get vaccinated (30, 31). Similar reasons for vaccine-hesitant behavior among people in secure living situations were identified in international studies (32). Regarding determinants influencing vaccine uptake and access to the vaccine among PEH, a few studies have been conducted. Existing studies with a focus on PEH from Italy (33), France (34), and the United States (35) aim mostly at vaccine acceptance rates. Only a few studies address determinants for vaccine-hesitant attitude or access to the vaccine (36–38). To our best knowledge, no study explored such topics among PEH in Germany until today.

This study aims to gain insights into thoughts and experiences on access and acceptance of COVID-19 vaccination among PEH to inform policymakers and service providers. Thus, we had the following research question: What factors influence the access to and acceptance of the COVID-19 vaccine among PEH in Berlin?

## 2. Materials and methods

In reporting our methods, we followed the Consolidated Criteria for Reporting Qualitative Research (COREQ) (39) and the Standards for Reporting Qualitative Research (SRQR) (40).

### 2.1. Study design

To understand which determinants influenced the accessibility of the COVID-19 vaccination and its acceptance among PEH living in Berlin, we designed a qualitative study with semi-structured interviews methodologically orientated on Mayring’s qualitative content analysis (41, 42). The qualitative study design was chosen because PEH have been under-researched so far and we had to assume that categories from frameworks may not be transferable to this group. In general, we aimed at identifying determinants of access and acceptance to COVID-19 vaccination, and in doing so, to test whether the models mentioned below are applicable to this.

Therefore, the interview guideline was developed theory-based by DS using the “5\u00B0C psychological antecedents of vaccination” by Betsch (29) and the “Theory of Access” by Saurman (19). We did not take the questions directly from the frameworks but designed them more openly to be open to determinants outside of the frameworks. The interview guide was developed in German, Polish, and English and included open-ended questions about the persons experiences with access to the COVID-19 vaccination and factors influencing their motivation to get the vaccine. A selection of sample questions is provided in Table 1.

**Table 1:** Selection of interview guide questions.

Icebreaker
What to you think of when I mention the COVID-19 vaccination?
Acceptability of the COVID-19 vaccine
What motivated or stopped you to from getting vaccinated when you first heard of the COVID-19 vaccine?
Access to the COVID-19 vaccine
What made it easy or hard for you to get the COVID-19 vaccine?

### 2.2. Recruitment of participants

As PEH are often harder to reach or to find than people not experiencing homelessness, we chose to approach PEH in person and ask them about their availability for an interview. To include homeless and shelterless people (both included in the term PEH), we approached aid providers in emergency and temporary day or night shelters in Berlin. Six providers accepted our request to conduct our study in their facilities during opening hours. To ensure a safer environment during the interviews, we conducted the interviews inside facilities instead of interviewing on the streets. We performed convenience sampling at first approach and adopted a purposive sampling strategy further on, aiming for a heterogeneous sample (maximum variation sampling) in terms of sex, language, and attitude toward COVID-19 vaccination. Researchers were not previously known to the interviewed persons.

### 2.3. Data collection

The guideline-based interviews were conducted between August 2021 and April 2022. In total, 19 interviews with 20 interviewees were carried out. One interview was conducted with two PEH at once because of the personal preferences of the interviewees. All other interviews were carried out with one person.

Due to limited space in most facilities, only a part of interviews was held in private rooms. The others were conducted in common areas such as dining or sleeping rooms in presence of non-participants. To ensure privacy and a secure interview setting we carried out the interviews in private area in the communal rooms. If necessary, nearby PEH were asked to leave the interview area for the duration of the interview to provide privacy.

In the concrete interview setting, the interviewer first informed about the study and asked for written consent for participation and the audio recording from the interviewees. Additional field notes on paper were made if necessary. Interviews were carried out until data saturation was achieved. The researchers have recognized point of data saturation in joint discussion. Four interviews were transcribed verbatim by DS, all the remaining were transcribed verbatim by JG according to previously set transcription rules based on the simplified transcription rules by Dresing and Pehl (43). Reflection and interpretation took place through the supervision of AS and discussion of preliminary results in interdisciplinary researcher’s workshops at the Institute of General Practice and Family Medicine and Institute of Medical Sociology, Epidemiology and Health Economics at Charité Berlin.

All interviews were conducted by DS, MK, and JG: five by DS in German or English, two by MK in Polish, and twelve by JG in German, English, or Polish. DS is a male medical student. MK and JG are female medical students, both with Polish backgrounds. Primary responsible for data collection processes after the first interviews held by DS and MK and data analysis was JG. The study was accompanied and supervised by AS, WH, and AL. All researchers had training in qualitative study processes. AS and WH are engaged in a researchers’ network working on health and homelessness. DS and JG were both engaged in the Berlin city mission during the process of the study; DS as the medical volunteer’s coordinator, and JG as a medical volunteer.

### 2.4. Data analysis

For data analysis, we used Mayring’s qualitative content analysis method. Using this method, we categorized the collected data and analyzed it subsequently (41, 42). Mayring’s qualitative content analysis allowed an analysis of mechanisms and determinants of vaccination access and acceptance in PEH. The choice of method was adapted to the research project and discussed with the participating researchers in advance of the analysis.

Categories were developed inductively from the data to reduce masking of unexpected findings and afterward revised in a deductive manner using the theoretical frameworks mentioned above. The first draft of the codebook was developed using five transcripts and then discussed with an interdisciplinary group of researchers. Suggestions for modifications were implemented and the further developed codebook was tested on other transcripts. JG coded all 19 interviews twice to account for continuous iterative adaptation of the codebook, AS counter-coded two interviews and another doctoral student counter-coded three interviews. Again, suggestions for modifications were discussed and integrated with mutual consent.

To explore the credibility of the findings, JG conducted member checking. JG discussed preliminary results with twelve non-interviewed PEH during a PEH self-advocacy meeting. The preliminary findings were discussed in an open group evaluation (44). The importance of certain themes was underlined and suggestions for further data analysis were made by the participating PEH. This helped us focusing the analysis on issues that were considered as very important by the PEH participating in the discussion. We chose to include this procedure because it was not possible to reach out to the previously interviewed PEH. Further measures to enhance credibility of the findings included ongoing discourse on methods and main focuses of the analysis with other researchers.

Transcription of interviews, Coding and Data analysis was carried out using the qualitative data analysis software MAXQDA. The transcripts were not translated for analysis and the codebook was developed in German. For this article, JG translated non-English quotes into English.

The analytical lens of our analysis was to focus on inhibiting and promoting determinants of access and acceptance of COVID-19 vaccination among PEH in Berlin.

### 2.5. Ethics statement

The study received ethical approval from the Charité ethics committee (Number = EA2/168/21). Data safety was performed according to the current data safety regulations at Charité.

All interviewees provided informed written consent. Interviews were pseudonymized in the transcripts, full names were not recorded intentionally and identifying aspects were paraphrased according to their function.

All participants included in this study were assessed as sane and oriented by the interviewer. In some cases, this also applied to participants who had possibly consumed mind-bending substances such as alcohol prior the interview. In the specific setting, the interviewer asked whether the participant considered him or herself able to participate in the interview with a brief conversation and explored if the participants assessment matched with the impression of the interviewer. PEH who were unwilling or unable to participate were not included in this study. The consumption of alcohol and other substances is prohibited in all facilities visited, however consumption of mild stupefacient’s of the participants cannot be excluded. The decision to include PEH who could give consent but might have consumed alcohol or other substances was taken to prevent selection bias toward potentially healthier PEH.

## 3. Results

We carried out 19 interviews with 20 participants. The length of the interviews was between 13 to 88 min with a mean of 29 min. Of the 20 participants, 75% were male, the mean age was 55 years (IQR. 25 P –75 P) and 75% have received at least one COVID-19 vaccination. Interviews were carried out in German (50%), Polish (20%), and English (30%). For a detailed overview of the participant’s sociodemographic data, vaccination status, and interview language please consider Table 2.

**Table 2:** Sociodemographic data of interviewees (*N* = 20).

		*n*
Sex	Male	15
	Female	5
Age	< 30 years	2
	30–50 years	6
	> 50 years	12
COVID-19 vaccination status	Vaccinated^*^	15
	Not vaccinated	5
Interview language	German	10
	Polish	4
	English	6
Country of origin	Germany	6
	Poland	3
	Russia	2
	USA	1
	Great Britain	1
	Romania	1
	Italia	1
	Namibia	1
	Bulgaria	1
	Netherlands	1
	Mazedonia	1
	Ireland	1

*At least one COVID-19 vaccine received.

The interview analysis focused on factors that contribute to understanding vaccination behavior and access to the COVID-19 vaccine among PEH. A deeper understanding of those aspects is relevant for future public health measures and better inclusion of PEH.

Table 3 shows a summary of inhibiting and enhancing factors for the acceptability of and access to the COVID-19 vaccine (Table 3).

**Table 3:** Summary of categories.

Acceptability of the COVID-19 vaccine	
Inhibiting factors	Fear of side effects caused by the vaccine
	Mistrust in politics and the health system
Enhancing factors	Trust in the safety and effectiveness of the vaccine
	High-risk perception of COVID-19
	Protecting others from transmission
	Taking part in daily life
Access to the COVID-19 vaccine	
Inhibiting factors	Need for personal documents or health insurance for a vaccine appointment
	Language barriers
Enhancing factors	Information and assistance in getting the vaccine by facilities for PEH
	Unbureaucratic vaccination offers

### 3.1. Acceptance of the COVID-19 vaccine

We explored the acceptance of the COVID-19 vaccine among PEH using Betsch’s 5C model (29). Overall, we found a high willingness to get vaccinated with the COVID-19 vaccine among most of our participants. This indicates a general acceptance of the vaccine as preventive measure.

*“I indeed think the vaccination is good.”* (T7, male, 52 years).

Not everyone showed that willingness to get vaccinated: a few participants refused to accept the vaccine under any condition.

*“I say it right away: I will never take this vaccine.”* (T12, male, 42 years).

#### 3.1.1. Confidence in the safety and efficacy of the COVID-19 vaccine

The degree of acceptance toward COVID-19 vaccination of some participants was dominantly influenced by their trust or mistrust of the vaccine. Especially the perception of the safety and effectiveness of the vaccine affected our participant’s vaccination attitude.

Some participants expressed their trust in the vaccine and did not experience side effects which enforced their trust in this preventive measure.

*“They say about Johnson & Johnson, you can have side effects. Honestly, I didn't have any either. I felt even better afterward. […]So, I can't understand the others why they [didn't do vaccination], it's not so bad.”* (T3, male, 44 years).

Trust in the vaccine was also expressed by referring to the high safety and effectiveness of COVID-19 vaccines.

*“Astra Zeneca and BioNTech […], Johnson & Johnson and Moderna, they are all at 90%. That is highly effective.”* (T1, male, 84 years).

Other participants expressed concerns regarding safety and effectiveness. Worries or misconceptions about different short- or long-term side effects of the vaccine enhanced the mistrust in the vaccine itself.

*"So, after the second injection I had extreme side effects […]. But I'd say that if it's gene-modifying, then it doesn't happen overnight, then somehow it can be harmful for years.”* (T14, female, 38 years).

PEH are especially exposed to side effects such as fever and possibly must deal with them in extremely vulnerable situations while living on streets or in unstable shelters.

*"I'm afraid of the side effects. […] Imagine I get vaccinated tomorrow, I'm here and then I have to go out during the day and I'm lying in bed with a fever like this*.” (T11, male, 24 years).

#### 3.1.2. Confidence in the political and healthcare system

Additional to the previous factors influencing confidence in the COVID-19 vaccine, some of the interviewed PEH mentioned aspects concerning politics and the healthcare system. Trust or mistrust in politics or the healthcare system can be interpreted from many statements. While some interviewees trusted the process of vaccine production and the involved healthcare system, others were skeptical.

*"No, I trust the scientists. They did not do that many years of school to kill me, did they?"* (T3, m, 44 years).

Reasons for not trusting the healthcare system in producing the vaccine were lack of time for proper research, assumptions on life- or health-threatening ingredients, and worries because of the development phase of the vaccine.

*“Besides, this time for testing was too little, that's why I say: "lab rabbit" because normally a drug is tested for 10 years before it gets the "ok" at all.”* (T6, female, 49 years).

Another aspect leading to less trust in the healthcare system for some participants was their experience of stigmatization while seeking out help.

*“If you go to a hospital, if you go to an emergency center and you say you have no permanent residence, then they act as if you are looking for an emergency overnight stay and do as little as possible at first.”* (T10, female, 57 years).

Some participants considered the entire health care system untrustworthy and criticized the heavy commercialization of the health care system.

*“So complete trust in medicine […] is not really there for me anyway. […] I don't want to say that medicine is completely wrong, but I also know that the pharmaceutical industry makes a lot of money with pills and everything else.”* (T14, female, 38 years).

When it comes to politics, interestingly most of the participants expressed mistrust in either individual politicians or the whole system itself. This distrust of politicians and the political system, as well as a general skepticism, led to a hesitant attitude toward vaccines.

*“So, if I let my trust in politics or politicians determine my vaccination decision, I wouldn't get vaccinated at all. […] I would like to see information that is more independent of political moods and elections and things like that and how many doses of vaccine are there right now and what the variants are.”* (T10, female, 57 years).

Especially the communication of politicians gained negative attention and was linked to unreliability.

*“This contradictory [name of politician] says this and that yes and every federal state [something different]. That worries me. What is it actually about? Germany can talk to us in straightforward language, can’t it?”* (T7, male, 52 years).

#### 3.1.3. Complacency

The need for vaccination depended on the COVID-19 risk perception and the assessment of the person’s resistance capacity.

*“Terrible, I got COVID, so, and I am in shape. […] And my immune system has always been good. But by God, COVID. […] it attacked me with a vengeance. You know, flat out. […] So, this is why I do not understand these people who do not get vaccinated.”* (T2, male, 63 years).

Others, however, did not feel at risk because of COVID-19 and therefore saw no need for a vaccine.

*“How dangerous do I think it [COVID-19] is? Actually, not really dangerous. I think [my body is protected] quite well [also without the vaccination].”* (T14, female, 38 years).

#### 3.1.4. Collective responsibility and protection through COVID-19 vaccination

When trusting the effectiveness of the COVID-19 vaccine, some of the interviewed PEH aimed to protect themselves or others by getting vaccinated. For some participants not only their own protection played a role in the vaccination decision but also the protection of others. This behavior indicates a collective responsibility toward the community in protecting each other through accepting preventive offers such as the COVID-19 vaccine.

*“When I'm sick […], I want to protect others so that I don't infect them. And that they then infect other people. […] you don't want to spread it.”* (T9, male, 55 years).

#### 3.1.5. Calculation

The acceptance of the COVID-19 vaccine depended strongly on the individual benefit–risk weighing. One aspect that helped some participants in accepting the vaccine was their desire to take part in daily life. Many daily life activities were restricted due to infection control measures and sometimes required a COVID-19 vaccination or a current antigen test.

*"So, I wanted to have my peace, I wanted to participate everywhere, I didn't want to be excluded, […] I want to get into every shop."* (T20, male, 62 years).

Therefore, the hope to reduce necessary COVID-19 tests because of prior vaccination was another motivator for some participants.

*“Here today everyone was standing in line, [to get tested in their noses]. I got the vaccine, they left me in peace.”* (T4, male, 59 years).

Interestingly, some participants were skeptical concerning the benefits and the need for the COVID-19 vaccine compared to other vaccines.

*“I'm skeptical […]. I'll put it this way: there are vaccinations that people need [like against] tetanus, […] or polio or something like that. But in general, with COVID-19 [vaccines] I am not sure.”* (T14, female, 38 years).

In general, it was important for some participants to inform themselves about the COVID-19 vaccine before getting vaccinated.

*“I would like to inform myself about […] what happens when I have recovered? And where does the protection come from and how long will it last - when does it wear off? […] I'd like to get more objective information about that.”* (T10, female, 57 years).

#### 3.1.6. Constraints

Constraints regarding the COVID-19 vaccine were often linked to factors that inhibited the accessibility of the vaccine in general. An in-depth discussion of such factors can be found below. For some participants, their daily life circumstances and barriers because of experiencing homelessness made it difficult to get vaccinated.

*“I couldn't do [the COVID-19 vaccine] because I was on the street. […], I slept on the benches and lost my health card and my ID. And I couldn't go anywhere because you need proof of who you are.”* (T3, male, 44 years).

### 3.2. Access to the COVID-19 vaccine

To understand perceptions and experiences of access to the COVID-19 vaccine among PEH, we based our analysis on Saurman’s Theory of Access (19).

#### 3.2.1. Availability and awareness of needs

The interviewed PEH mostly perceived support and assistance from facilities for PEH as very helpful. Facilities for PEH were aware of the special needs of PEH and some offered information on COVID-19 vaccines or appointments for a vaccine. Those services were additionally offered to the usual services like providing food and a place to sleep. Some participants underlined the importance of the employees and volunteers in facilities for PEH. If they are considered trustworthy, they can play an important role in some of the participant’s decision-making.

*“[I would have] rather not [taken the vaccine without information of people working in this night shelter], because I wouldn't have known where to go and stuff like that. And here they came, they came beautifully, nicely "do you want it?". We had a normal conversation because the Polish woman was also going around. They said, "do you want the vaccine?" and I said, "of course, yes". Normally, no.”* (T17, female, 56 years).

In addition to the information provided by staff and volunteers working in facilities for PEH, some PEH emphasized the importance of addressing language barriers.

*“The problem here for foreigners is language.”* (T3, male, 44 years).

The inability to understand information about the vaccine was in line with lower acceptance rates. Therefore, translation by staff and volunteers at facilities was found to be very helpful.

*“[Information is important for me, then one can learn.] And especially since there is another Polish woman working here, she will always translate.”* (T17, female, 56 years).

#### 3.2.2. Accessibility and affordability

In terms of accessibility, many PEH considered COVID-19 vaccination offers from facilities for PEH as enhancing access to the vaccine. Many referred accessing the vaccine within such offers as easy and convenient.

*“Now I came to the [name of provider for PEH], they made it easier for me to do it. They told me [about the vaccine,], I did it, that was the fastest. Before I couldn't do it because I was on the street.”* (T3, male, 44 years).

Compared to most participants’ very positive opinions on vaccination offers assisted by facilities for PEH, their perception of public vaccination offers differed strongly. A few gave a positive response to public vaccination offers in big vaccination centers and pointed out a good organization in Germany.

*“All I can say is that Germany has done such a wonderful job about this.”* (T2, male, 63 years).

Nevertheless, many participants indicated a panoply of different barriers in the context of public vaccination offers. Some PEH had a negative experience with offers that were difficult to reach and going there required high transportation costs.

*“What the price was - […] you know, it’s free, but almost 6 € I’ve got to pay for the metro ticket. I mean that’s a bottle of wine or a couple of drinks […]. Again, for the poor people, that is a lot of money.”* (T15, male, 59 years).

Secondly, some participants experienced a lack of appointments.

*“An appointment [would make it easier for me to get vaccinated].”* (T7, male, 52 years).

#### 3.2.3. Acceptability and adequacy of service

In terms of acceptability and adequacy of service, conditions of public vaccination offers were considered unbearable by some participants. They referred to long waiting times and hard reachability.

*“I twice went to [the COVID-19 vaccine center in] Tempelhof and once to the Messe Berlin. And you know, it takes me an hour to get there and then I have to wait in those inhumane conditions.”* (T15, male, 59 years).

Another aspect often perceived as a barrier to access to the COVID-19 vaccine was the need for identification documents for receiving a vaccine in public vaccination centers.

*“For the homeless, [it is often difficult], because [identification] documents are often stolen […] and if the vaccination fails because of that and then they say […] "You'll get an appointment in five months", well, congratulations!”* (T10, female, 57 years).

In summary, access to the COVID-19 vaccine was perceived as easier the lower the threshold. Many participants experienced low-threshold vaccination offers in facilities for PEH with a focus on the specific needs of PEH.

## 4. Discussion

Overall, the data indicate a positive attitude toward the COVID-19 vaccination among PEH. We were able to identify inhibiting and enhancing factors for the accessibility and acceptance of the COVID-19 vaccine. Interestingly, vaccine acceptance was found to be vaccine-specific: some participants rejected the COVID-19 vaccination while accepting other vaccines. We noticed this aspect outside of the theoretical frameworks. This observation matches studies indicating an overall decrease in vaccine confidence rates after the outbreak of SARS-CoV-2 in comparison to pre-pandemic times (32). In addition to general vaccine hesitancy, the COVID-19 vaccine also worried people with safety concerns because of the quick development or perceived lack of efficiency of the vaccine. Vaccine hesitancy against the COVID-19 vaccine was also associated with a low-risk perception of COVID-19 (45).

We were able to identify the dimensions of the 5C model (confidence, complacency, constraints, calculation, and collective responsibility) in our findings (29). Acceptance of the COVID-19 vaccine was predominantly influenced by trust in the vaccine itself or mistrust in the politics and healthcare system. Confidence in the safety or efficacy of the vaccine was associated with a greater willingness to get vaccinated, while mistrust or misconceptions about the vaccine was linked to hesitant behavior. Fear of side effects caused by the vaccine or misconceptions about the vaccine played a role in some participants’ vaccine attitudes. Interestingly, most participants clearly stated their mistrust of the political system. This mistrust, sometimes combined with mistrust of the healthcare system, was also associated with lower acceptance of the vaccine. Experienced stigmatization in the health and political system could be a reason for mistrust (15). A systematic review on improving vaccination rates in PEH also suggests providing clear and stringent information in order to tackle misinformation and mistrust (26). In accordance with our findings from this Germany-based study, prior international studies on the COVID-19 vaccine and PEH report safety concerns regarding the vaccine, distrust in the government, and vaccine manufacturers as enhancers for vaccine-hesitant attitude (36, 37). Similar results were also found in studies focusing on people in secure living situations (45). However, some participants, although distrusting the political or healthcare system, mentioned self-protection, protection of others, or the willingness to be part of social life as prevailing drivers to accept vaccination. Especially the willingness to be included into social life is an important aspect for PEH. Recent studies have shown that loneliness and social isolation among PEH can be considered a determinant for health (46, 47). The impact of loneliness on health is also well described for people in secure living situations (48). Considering that PEH are a marginalized group, this aspect is of great importance. Calculating risks and benefits and the willingness to prevent the spread of COVID-19 was also found among young PEH in a study from the United States. Interestingly, this study shows slightly lower vaccine acceptance which might be explained by the younger study population (38). Low-risk perception of COVID-19 was associated with vaccine-hesitant attitude among the participants of our study. A German study from 2021 indicated low fear of COVID-19 among PEH. They also described higher fear of COVID-19 among PEH aged 50 to 64 (49) which is the main age group in our cohort and might explain the mainly positive attitude toward the COVID-19 vaccination. That age might have a strong impact on the attitude toward vaccine attitude is also mentioned by a Danish study von COVID-19 vaccine coverage among PEH. PEH aged 18–24 years showed lower vaccination rates than older PEH (50). Constraints regarding the COVID-19 vaccine were often associated with other dimensions of acceptability as safety concerns or distrust in the political system. Overall, PEH who participated in our study perceived several life-circumstances-related barriers in accepting the vaccine, such as difficulties accessing public vaccination sites and everyday struggles that limited opportunities to get vaccinated.

To operationalize access to the COVID-19 vaccine, we were able to implement the Theory of Access (availability, accessibility, adequacy of service, affordability, acceptability, and awareness of needs) as the analytical lens in our study (19). COVID-19 vaccination offers by facilities for PEH were considered as very helpful and matched PEH needs in terms of availability and awareness. Personal interaction and engagement of staff with PEH facilitated willingness for vaccination by providing trusted information or simply by answering open questions. A similar outcome was reported in studies from the United States. Low-barriers for testing for COVID-19 and vaccine offers facilitated by community health outreach workers were associated with higher vaccine uptake and better accessibility (36, 37). In addition of the benefits personal interaction mentioned in our study prior research highlights the positive effect of offering vaccines at sites already frequently visited by PEH (26). This aspect is not as evident in our data, but still fits with the overall positive response to vaccination offers at facilities for PEH. Another important inhibiting factor in accessing the COVID-19 vaccine were language barriers. Addressing language barriers through the provision of information in a variety of languages can enhance the participation of PEH and are important aspects of improving access to healthcare for PEH (13, 51). In terms of accessibility and affordability, the support and assistance of facilities for PEH in organizing vaccination or an appointment were again found to be very helpful. This enhancing factor was acknowledged by almost every participant and helped many of them to receive their vaccine. In our study, unbureaucratic vaccination offers were also linked to better accessibility for PEH, as many struggle with personal documents such as an ID card. Some participants experienced the publicly available COVID-19 vaccination sites as disappointing because they were considered as too bureaucratic, lacked appointments, or were hard to reach. Vaccine offers at general practitioners’ practices were rarely mentioned among our participants, possibly because of avoidant behavior due to previous experiences of stigmatization in the healthcare system (15, 52).

There are several limitations to our study. The interviews were conducted at different periods due to organizational reasons and the seasonality of the shelters. Therefore, the circumstances of the COVID-19 pandemic as the availability of vaccines, current infection control measures, and political discourse differed. We also assume a selection bias toward PEH with positive vaccine attitudes and higher educated PEH, as our interview requests might have not caused interest by PEH with a negative attitude toward vaccinations or might have caused fears in less educated individuals. Furthermore, we did not include PEH who were unable to participate in the interviews because of substance abuse. This might have led to a selection bias toward healthier PEH. To address this limitation, we conducted maximum variation sampling based on theoretical criteria. The dimensions of access (19) are independent but interconnected. Therefore, the categorization of our participants’ statements was not always clear. Further, to increase the validity of our results we conducted interviews in German, English as well as in Polish. However, the fact that we only included three languages in our interview study is also a limitation. It should also be considered that not all interviewees were native speakers of the respective language. This also applies to the interviewers. This might have been an obstacle for participants to fully articulate their ideas and feelings. Because we conducted the interviews in facilities with limited space, we could not provide private rooms for each interview. We were always seeking highest degree of privacy. Sometimes, non-participants were present in adequate distance. This might have had an influence on the openness of statements made in the respective interviews. We conducted one of our interviews with two participants at once. This might have influenced their perception of the interview setting as a safe space to talk but also engaged them to think further on specific issues because of an ongoing interactive discussion. Through member checking with PEH community members (44) and representatives and interprofessional exchange with other researchers specialized in the subject matter and or the subject methods we aimed to increase validity and credibility of our results.

Building a multidimensional strategy to better reach PEH is also recommended by the *National Federation for the Homeless* (Bundesarbeitsgemeinschaft Wohnungslosenhilfe, BAG W). Specifically, vaccination sites are suggested at locations that are already visited by PEH to ensure better integration into daily life, better accessibility, and less discrimination or experience of stigma. Furthermore, target-group-specific information given in different languages was recommended (26, 52, 53). Recommendations for a better vaccine uptake include a stronger and clearer promotion, development of interventions and stakeholder collaboration as PEH themselves might be interested in opportunities in being involved in preventive strategies for their community (26). Further, participatory health promotion involving PEH have been shown to be appropriate, acceptable and effective for community-based interventions (54). Our study contributes to understanding determinants of access and acceptance for preventive public health measures such as the COVID-19 vaccination among PEH in urban areas in Germany. Our findings are in line with prior international studies and add information on Germany in this context. We were able to identify determinants of vaccination access and acceptance using the 5C model and Theory of Access. Overall, our study suggests that vaccination acceptance is mainly influenced by psychological factors and depends in part on access factors. Further, the political discourse around COVID vaccination has shown to play an important role.

The identified fields for action offer important opportunities to steer vaccination offers for PEH in the future.

For future preventive public health measures, interest groups strongly recommend adapting prevention strategies to the special needs of vulnerable groups in society, especially for PEH (53, 55). Therefore, establishing target-group-oriented health care and public health strategies are crucial. Policymakers have recognized this gap and called for structural changes aiming at openness, accessibility, and reduced discrimination in the healthcare system in Germany (56). However, concrete steps to bring this aim further are currently lacking.

## Data availability statement

The raw data supporting the conclusions of this article will be made available by the authors, without undue reservation.

## Ethics statement

The studies involving human participants were reviewed and approved by Charité - Universitätsmedizin Berlin, Ethics Committee (EA2/168/21). The patients/participants provided their written informed consent to participate in this study.

## Author contributions

JG, AS, and DS: study design. JG and DS: recruitment of participants. JG, DS, and MK: data collection. JG, AS, and MP: data analysis. JG: writing of original manuscript. JG, AS, and WH: revision and editing. AS, WH, and AL: supervision. All authors have read and agreed upon the published version of the manuscript.

## Funding

This study received financial support from the Open Access Publication Fund of Charite - Universitatsmedizin Berlin and the German Research Foundation (DFG).

## Conflict of interest

The authors declare that the research was conducted in the absence of any commercial or financial relationships that could be construed as a potential conflict of interest.

## Publisher’s note

All claims expressed in this article are solely those of the authors and do not necessarily represent those of their affiliated organizations, or those of the publisher, the editors and the reviewers. Any product that may be evaluated in this article, or claim that may be made by its manufacturer, is not guaranteed or endorsed by the publisher.

## Abbreviations

COSMO: COVID-19 Snapshot Monitoring
COVID-19: Coronavirus Disease 2019
COVIMO: COVID-19 Impfquoten Monitoring (COVID-19 vaccination rate monitoring)
PEH: people experiencing homelessness
