# Corrigendum: Determinants of COVID-19 vaccine acceptance and access among people experiencing homelessness in Germany: a qualitative interview study

**DOI:** 10.3389/fpubh.2023.1245524

**Published:** 2023-07-21

**Authors:** Julianna Grune, Darius Savelsberg, Marta Kobus, Andreas K. Lindner, Wolfram J. Herrmann, Angela Schuster

**Affiliations:** ^1^Institute of General Practice and Family Medicine, Charité - Universitätsmedizin Berlin, Corporate Member of Freie Universität Berlin, Humboldt-Universität zu Berlin and Berlin Institute of Health, Berlin, Germany; ^2^Institute of International Health, Charité - Universitätsmedizin Berlin, Corporate Member of Freie Universität Berlin, Humboldt-Universität zu Berlin and Berlin Institute of Health, Berlin, Germany

**Keywords:** homelessness, prevention, vaccine acceptance, vaccine hesitancy, vaccine access, COVID-19, primary care, access to health care

In the published article, there was an error. We noticed a spelling mistake in the description of a framework we used for our data analysis.

A correction has been made to the **section 2) Materials and methods, sub-section 2.1) Study design**, in the first sentence of the second paragraph. This sentence previously stated:

“Therefore, the interview guideline was developed theory-based by DS using the “5\u00B0C psychological antecedents of vaccination” by Betsch (29) and the “Theory of Access” by Saurman (19).”

The corrected sentence appears below:

“Therefore, the interview guideline was developed theory-based by DS using the “5C psychological antecedents of vaccination” by Betsch (29) and the “Theory of Access” by Saurman (19).”

The authors apologize for this error and state that this does not change the scientific conclusions of the article in any way. The original article has been updated.

